# SAPHO Syndrome Mimicking Infectious Spondylodiscitis and Bone Metastasis

**DOI:** 10.1155/2021/5577257

**Published:** 2021-09-04

**Authors:** S. Biuden, K. Maatallah, H. Riahi, H. Ferjani, M. D. Kaffel, W. Hamdi

**Affiliations:** ^1^Department of Rheumatology, Charles Nicolle Hospital, University of Tunis El Manar, Tunis, Tunisia; ^2^Department of Rheumatology, Kassab Institute, University of Tunis El Manar, Tunis, Tunisia; ^3^Department of Radiology, Kassab Institute, University of Tunis El Manar, Tunis, Tunisia

## Abstract

The acronym SAPHO (synovitis, acne, pustulosis, hyperostosis, and osteitis) includes diseases with similar osteoarticular manifestations and skin conditions. Making this diagnosis is not always obvious, especially when the clinical presentation does not fit the typical pattern of the disease or it occurs in a particular field. We described three cases where the diagnosis was difficult. A 46 year-old woman presented with cervical pain. The cervical X-ray showed the aspect of an ivory C5 vertebra. The patient had, however, preserved general condition, no signs of underlying neoplasia, nor other joint complaints. Blood analysis was normal. Tomography did not find any suspect lesion but showed sclerosis and hyperostosis of the manubrium. Scintigraphy showed the characteristic “bullhead” appearance. A 61-year-old woman had thoracic and lumbar pain. MRI showed spondylodiscitis in D3-D4, D4-D5, D5-D6, D6-D7, and L1-L2 with paraspinal soft tissue involvement, simulating infectious spondylodiscitis. Infectious investigations and discovertebral biopsy performed twice were negative. SAPHO syndrome was then suspected. Bone scintigraphy showed uptake in the chondrosternal articulations and D4 to D7 vertebrae. The diagnosis of SAPHO was established. The third case was a 46-year-old man with a lung adenocarcinoma. Staging for metastatic disease, a TAP tomography was performed and showed osteosclerosis of D8 to D12 and intra-articular bridges in the sacroiliac joints. MRI and scintigraphy eliminated malignancy and confirmed the diagnosis of SAPHO. In our cases, imaging findings could facilitate differentiating SAPHO syndrome from other diseases.

## 1. Introduction

The acronym SAPHO (synovitis, acne, pustulosis, hyperostosis, and osteitis) includes a group of diseases with similar osteoarticular manifestations and skin conditions [1, 2]. The incidence is thought to be less than 1/10000, with the highest occurrence in children and young adults [3, 4]. There are no validated diagnostic criteria, and the diagnosis relies on clinical and radiological findings. When the clinical presentation did not fit the typical pattern of the disease or occur in a particular field, distinguishing SAPHO from other diagnoses, especially bone tumor and infectious disease, may be difficult. Herein, we present different cases where the diagnosis was not obvious.

## 2. Cases' Description

### 2.1. Case 1

**Figure 1:**
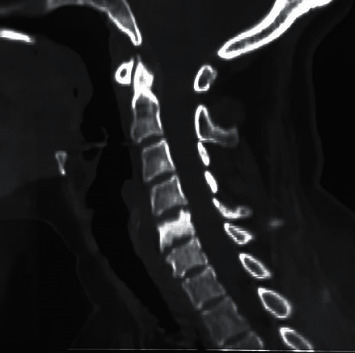
Sagittal reconstruction (bone window) shows the aspect of an ivory C5 vertebra.

**Figure 2:**
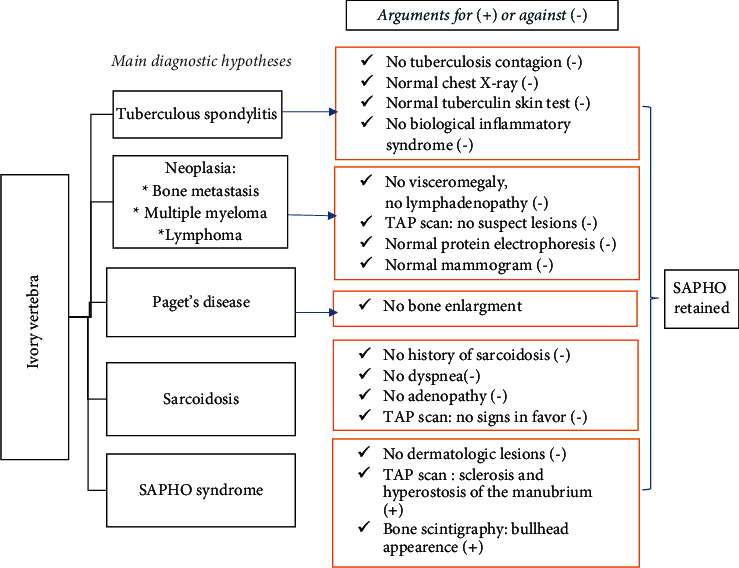
Summary diagram of case 1 diagnosis.

A 46-year-old woman presented with an eight-month history of cervical pain. She had no personal medical history and no history of retinoid intake. She had selective pain upon palpation at the cervical spine. The patient had preserved general condition, no signs of underlying neoplasia or infection, nor other joint complaints. She had neither visceromegaly nor lymphadenopathy. There was no biological inflammatory syndrome, her C-reactive protein (CRP) was 2 mg/l, and her erythrocyte sedimentation rate (ESR) was 20 mm. The blood count and the phospho-calcium balance were normal. The cervical spine X-ray revealed osteosclerosis of the C5 vertebral body with the aspect of an ivory vertebra which was confirmed on the cervical scanner (Figure 1). The spinal magnetic resonance imaging (MRI) showed an abnormal signal on C5 and C6 with a hypointense signal on T1 and T2 and a hyperintense signal on T2 short TI inversion recovery (STIR) and postcontrast enhancement. In front of the imaging findings and even though the lack of signs was suggestive of neoplasia, bone metastasis was the most likely diagnosis. Further investigations were required to screen for primary neoplasia. The mammography and the cervical ultrasound were normal. The thoraco-abdominopelvic (TAP) scan did not show any suspect lesion but revealed sclerosis and hyperostosis of the manubrium and fusion on edges of sternocostal joints. There was no sacroiliitis. We should note that the patient did not have a history or current chest wall pain. The bone scintigraphy showed increased uptake in the sternoclavicular articulations with a characteristic “bullhead” appearance. As infection and neoplasia were rolled out and as our patient fulfilled Benhamou et al.'s [2] diagnostic criteria, the SAPHO syndrome diagnosis was established (Figure 2). Treatment with diclofenac was initiated, with a real clinical improvement.

### 2.2. Case 2

**Figure 3:**
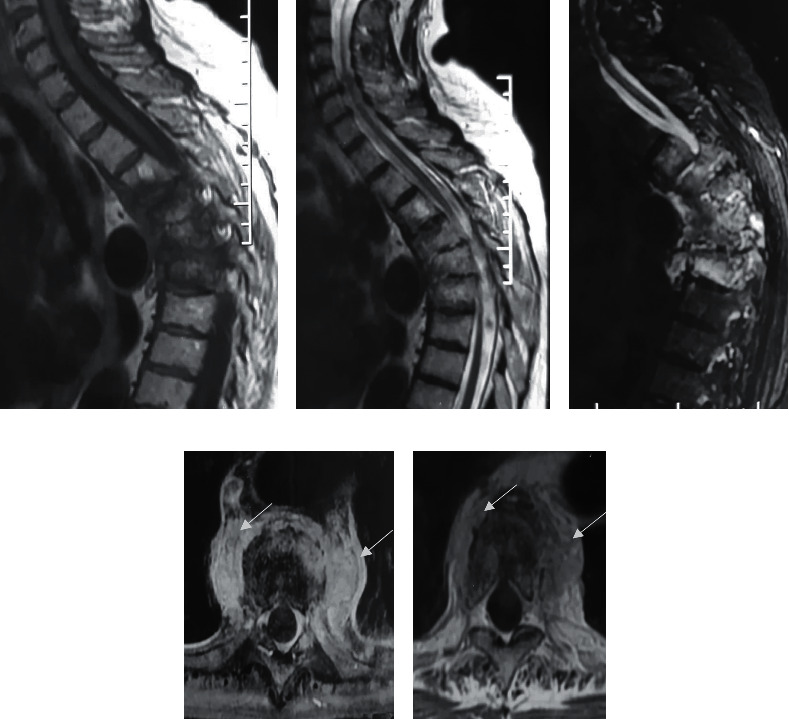
Magnetic resonance imaging. (a) Sagittal T1 WI, (b) sagittal T2 WI, and (c) sagittal STIR WI showing irregularity of the end plates from D4 to D7, high T2 signal in the disc, marrow signal change in the adjacent vertebra (T1 low and T2 high), and soft tissue mass (arrow). (d, e) Magnetic resonance imaging axial T1 after contrast showing soft tissue microabscess (arrow).

**Figure 4:**
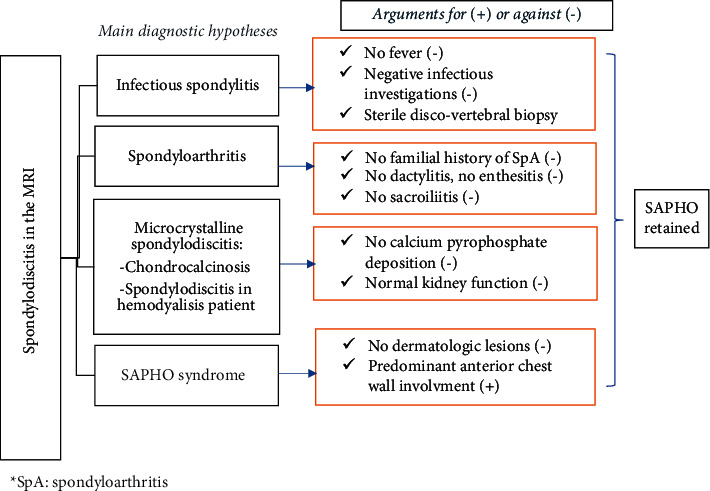
Summary diagram of case 2 diagnosis. SpA: spondyloarthritis.

**Figure 5:**
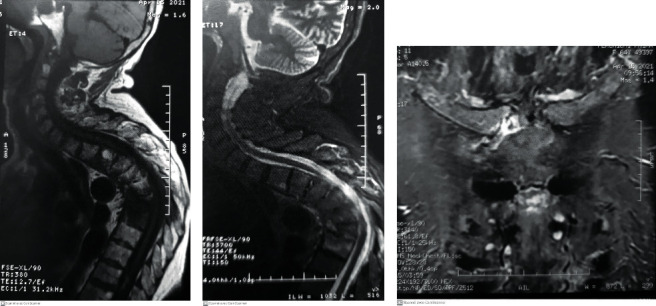
(a–c) Inflammatory marrow signal from T1 to T9 complicated by kyphosis at the top of T7 and associated with inflammatory damage at the anterior chest wall, predominantly the right sternoclavicular joint.

A 61-year-old woman presented with inflammatory thoracic and back pain. Clinical examination showed a stiffness of the lumbar spine. She had no fever. The CRP and ESR were elevated to 14 mg/L and 83 mm, respectively. Conventional radiography showed a narrowing of the intervertebral discs of D5-D6 and D6-D7. Spine MRI showed spondylodiscitis in D3-D4, D4-D5, D5-D6, D6-D7, and L1-L2 and soft tissue involvement, simulating infectious spondylodiscitis (Figure 3). All infectious investigations were negative: chest X-ray, cytobacteriological urinalysis, tuberculin skin test, test for mycobacterium tuberculosis in sputum and urine, and Wright's serological test. The discovertebral biopsy showed unspecific inflammation. Despite the negativity of the infectious investigation and given MRI findings and the notion of unpasteurized milk consumption, the diagnosis of brucellar spondylodiscitis was first evoked, and treatment with Rifadin and doxycycline was decided upon. After six months of treatment, no clinical improvement was noted. CRP and ESR remained elevated. No bone reconstruction was noticed in the X-rays. So, a second MRI was performed and showed the stabilization of the original lesions and the appearance of a new lesion in L4-L5. A second infectious investigation was performed but was negative. A discovertebral biopsy was indicated and was sterile. The diagnosis of SAPHO was then suspected, and bone scintigraphy was performed showing uptake in the chondrosternal articulations and D4 to D7 vertebrae, supporting the diagnosis of SAPHO (Figure 4). Treatment with NSAIDs associated with brace wearing was indicated. The patient did not respond to four classes of NSAIDs, so the decision was to switch to etanercept with initial clinical and biological improvement and unchanged lesions in MRI after 3 months. The treatment was received for 1 year. The treatment was received for 1 year without any efficiency. A new MRI was then performed showing an inflammatory marrow signal from T1 to T9 complicated by kyphosis at the top of T7 and associated with inflammatory damage at the anterior chest wall, predominantly the right sternoclavicular joint (Figures 5(a)–5(c)). Switch to infliximab was then indicated.

### 2.3. Case 3

**Figure 6:**
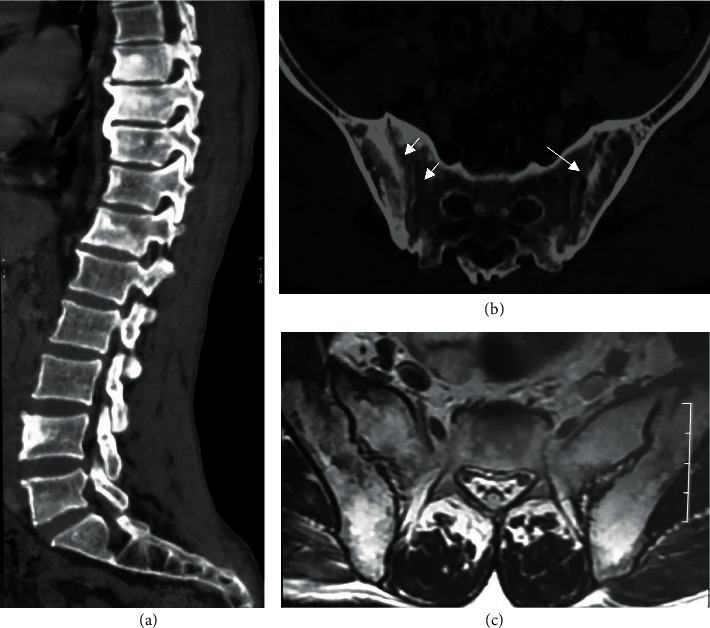
(a) Sagittal CT image showing osteosclerosis of vertebral bodies of D8 to D12 and L4 with paravertebral ossifications. (b) Axial CT image showing intra-articular bridges and multiple well-defined erosions with sclerotic margins. Bone sclerosis is blurred within the trabecular bone. (c) Magnetic resonance imaging axial T1 WI showing bilateral sacroiliitis with a fatty marrow signal.

**Figure 7:**
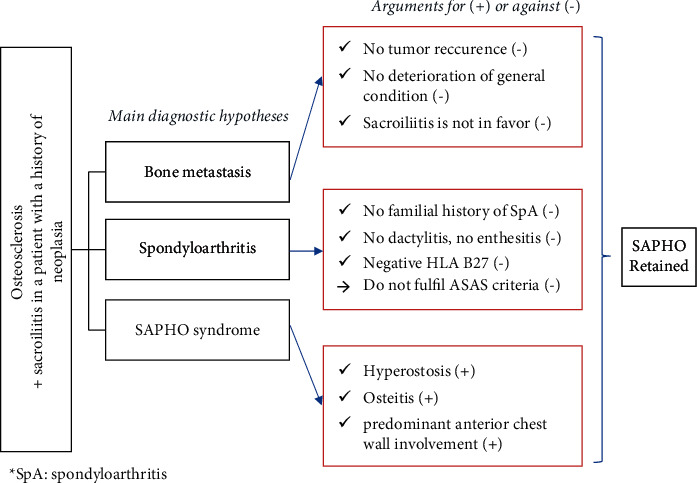
Summary diagram of case 3 diagnosis. SpA: spondyloarthritis.

**Figure 8:**
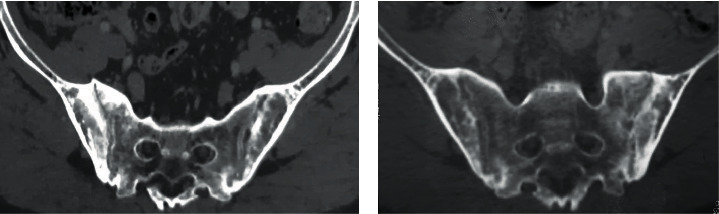
CT scan 12 months later showing ankylosis of the sacroiliac joint.

A 46-year-old man was followed in the oncology department for lung adenocarcinoma. The TAP scan, carried out as part of the extensive assessment of the disease, showed osteosclerosis of vertebral bodies of D8 to D12 and L4 with paravertebral ossification (Figure 6(a)) and bilateral osteosclerosis in the sacroiliac joints with intra-articular bone bridges (Figure 6(b)). The patient reported a history of mechanic low back pain lasting for 20 years but no current flare-up. Even though intra-articular sacroiliac bone bridges could not be explained by bone metastasis, given the history of neoplasia, this was the first suspected diagnosis. The patient underwent an MRI that showed bilateral sacroiliitis (Figure 6(c)) and an abnormal signal in the D4 to D11 vertebrae. Human leukocyte antigen (HLA B27) was negative. Bone scintigraphy showed increased uptake in the manubrium, D8 to D11 vertebrae, sacroiliac joints, right collarbone, and skull. Given that the tumor was in remission and there was no recent worsening of the low back pain and considering the intra-articular sacroiliac bone bridges and the involvement of the anterior chest wall, the diagnosis of SAPHO syndrome was established (Figure 7). Treatment included diclofenac. A control TAP scan was performed, showing stable spine lesions and ankylosis of the sacroiliac joint (Figure 8).

## 3. Discussion

SAPHO syndrome was first described by the French Society of Rheumatology in 1987 as a group of musculoskeletal manifestations that may or may not be associated with dermatologic lesions [1]. The osteoarticular manifestations include synovitis, hyperostosis, and osteitis. The skin manifestations are typically palmoplantar pustulosis and acne. They can either precede (40–60%), occur simultaneously (30%), or occur after the start of the osteoarticular lesions (32–60%) [5, 6]. At least 15% of adult patients may never have skin manifestations. The diagnosis of SAPHO syndrome is obvious when the clinical presentation fits the typical features of the disease. The diagnosis can be confusing if atypical sites are involved, there is no skin disease, or it occurs in a particular field. In our cases, no patient had dermatologic manifestations. The osteoarticular involvement is commonly insidious at onset [7], like in our third patient where the diagnosis was fortuitous. Although the sternoclavicular involvement is the most common localization, other bones in the axial or peripheral skeleton can be involved. The spine is the second most common site in the disease being involved in up to one-third of cases, most frequently in the thoracic spine, followed by the lumbar and cervical spine [8]. Spinal involvement is usually segmental, most commonly affecting a single vertebra, but occasionally affects up to four lesions [8]. All our patients had over one vertebra affected, and our third patient even had five vertebrae affected. There is frequent involvement of the soft tissues, but abscess and epiduritis are not common [8]; however, our second case presented liquid paravertebral masses, which, to the best of our knowledge, have not been reported before. The presence of liquid masses makes the differential diagnosis with an infectious disease more challenging, especially knowing that an infectious hypothesis involving *Proprionibacterium acnes* has been proposed [9]. *P. acne* activates innate immune response through toll-like receptors [10]. It could persist in a latent form in bone cells and enhance IL1*ß* production by skin and bone cells leading to dermatologic and osteoarticular lesions [11]. Sacroiliitis of SAPHO occurs in 13% to 52% of cases [12, 13]; it is often unilateral and is characterized by hyperostosis and sclerosis, especially in the iliac side [7]. Our third patient had bilateral sacroiliitis. HLA B27 has been reported to be positive in 13–30% of SAPHO patients [14]. In our third patient, it was negative. MRI is the most sensitive imaging technique in evaluating soft tissue swelling, synovial reaction, and intra-articular effusion and synovial reaction in SAPHO syndrome [15]. Bone scintigraphy can be very useful because it not only shows increased uptake in the affected sites but also reveals silent lesions [16]. The most frequent localization is the anterior chest wall, especially the characteristic appearance of the bull's head sign, as seen in the first case. Bone biopsy does not reveal specific lesions, but it helps to exclude malignant and infectious etiologies [7]. Most patients are treated symptomatically with nonsteroidal anti-inflammatory drugs (NSAIDs) and analgesics. The role of antibiotics remains unclear. Conventional disease-modifying antirheumatic drugs (DMARDs) are a reasonable choice after NSAIDs' failure. In the refractory cases, anti-TNF-alpha agents are used. A good response is frequently obtained like in our second case [17]. The prognosis of SAPHO syndrome is considered to be relatively good [12]; however, in our second case, the disease was particularly severe. The diagnostic delay, because of the lack of awareness of the syndrome, is a major source of unnecessary and invasive explorations.

In conclusion, the diagnosis of SAPHO syndrome is obvious when typical bone lesions are associated with typical skin manifestations. The diagnosis is much more challenging when patients are free of skin disease or when it occurs in a typical field. In these cases, imaging findings could facilitate differentiating SAPHO syndrome from other diseases, hence the need for greater awareness of the radiological features of the disease.
